# Emotion Classification from Multi-Band Electroencephalogram Data Using Dynamic Simplifying Graph Convolutional Network and Channel Style Recalibration Module

**DOI:** 10.3390/s23041917

**Published:** 2023-02-08

**Authors:** Xiaoliang Zhu, Gendong Liu, Liang Zhao, Wenting Rong, Junyi Sun, Ran Liu

**Affiliations:** 1 National Engineering Research Center of Educational Big Data, Central China Normal University, Wuhan 430079, China; 2 Engineering Product Development Pillar, Singapore University of Technology and Design, Singapore 487372, Singapore

**Keywords:** EEG, emotion classification, graph neural network, SRM, channel selection

## Abstract

Because of its ability to objectively reflect people’s emotional states, electroencephalogram (EEG) has been attracting increasing research attention for emotion classification. The classification method based on spatial-domain analysis is one of the research hotspots. However, most previous studies ignored the complementarity of information between different frequency bands, and the information in a single frequency band is not fully mined, which increases the computational time and the difficulty of improving classification accuracy. To address the above problems, this study proposes an emotion classification method based on dynamic simplifying graph convolutional (SGC) networks and a style recalibration module (SRM) for channels, termed SGC-SRM, with multi-band EEG data as input. Specifically, first, the graph structure is constructed using the differential entropy characteristics of each sub-band and the internal relationship between different channels is dynamically learned through SGC networks. Second, a convolution layer based on the SRM is introduced to recalibrate channel features to extract more emotion-related features. Third, the extracted sub-band features are fused at the feature level and classified. In addition, to reduce the redundant information between EEG channels and the computational time, (1) we adopt only 12 channels that are suitable for emotion classification to optimize the recognition algorithm, which can save approximately 90.5% of the time cost compared with using all channels; (2) we adopt information in the θ, α, β, and γ bands, consequently saving 23.3% of the time consumed compared with that in the full bands while maintaining almost the same level of classification accuracy. Finally, a subject-independent experiment is conducted on the public SEED dataset using the leave-one-subject-out cross-validation strategy. According to experimental results, SGC-SRM improves classification accuracy by 5.51–15.43% compared with existing methods.

## 1. Introduction

In recent years, with the development of artificial intelligence, emotion classification has presented important application prospects in human–computer interaction, disease monitoring, artificial intelligence education, intelligent transportation, and other fields. For example, if drivers’ emotions can be recognized, some intervention measures may be taken to avoid accidents when drivers’ concentration is severely disturbed [1]. Emotion classification methods are generally divided into two categories according to the types of signals analyzed: one is based on non-physiological signals, such as text, audio, facial expression, and body language; the other is based on physiological signals, such as electroencephalogram (EEG), electrocardiogram, galvanic skin response (GSR), and photoplethysmogram (PPG). Progress has been made in both types of methods. For example, Li et al. proposed a semi-supervised deep facial expression recognition method based on an adaptive confidence margin [2]. Kang et al. proposed a one-dimensional convolutional autoencoder to classify emotions by PPG and GSR [3]. In contrast, it is unfriendly to use non-physiological signals to identify emotions for special people or people with facial, limb, and voice damage caused by accidents. Physiological signals are not easily affected by these external factors, are difficult to conceal, and can reflect the real emotional state of an individual. Studies have shown that the central nervous system (brain) regulates and controls the autonomic nervous system in emotional processes [4]. EEG has the characteristics of being non-invasive, portable, reliable, and inexpensive. Therefore, the method of emotion classification based on EEG has significant advantages.

At present, many traditional machine learning methods have been proposed and applied to EEG-based emotion classification, but most of them rely on manual feature extraction, which affects their universality. With the extensive application of deep learning, EEG-based emotion classification has achieved remarkable success. For example, Du et al. proposed an attention-based long short-term memory (LSTM) with a domain discriminator (ATDD-LSTM) method to classify emotions based on multi-channel EEG signals. When the differential entropy (DE) feature from the SEED dataset was used as the input, the accuracy of a subject-dependent experiment reached 91.08%, whereas that of a subject-independent experiment reached 90.92% [5]. Common EEG signal features include time-, frequency-, and spatial-domain features. Traditional methods only consider a single feature or a combination of two features. Subsequently, considering the complementarity of features, researchers integrate multiple features such as spatial-, frequency-, and time-domain features and other features to obtain more emotion-related features of EEG. For example, Jia et al. proposed a spatial-spectral-temporal-based attention three-dimensional (3D) dense network (SST-EmotionNet) and designed parallel spatial-spectral stream and spatial-temporal streams to learn time-, spatial-, and frequency-domain features, achieving accuracy of 96.02% on the SEED dataset [6]. EEG signals are generally transformed from the time domain to the frequency-domain. The most commonly used method is to decompose EEG signals into five sub-bands (i.e., delta: 1–3 Hz; theta: 4–7 Hz; alpha: 8–13 Hz; beta: 14–30 Hz; gamma: 31–50 Hz) and then extract frequency features, including DE, power spectral density (PSD), and differential asymmetry (DASM) features [7,8]. Recently, researchers have realized that EEG is non-Euclidean data, so EEG is mostly represented in the form of a graph. For example, Song et al. proposed a dynamic graph convolutional neural network (DGCNN) for EEG-based emotion classification, which achieved 79.95% accuracy on the SEED dataset [9]. Other researchers have conducted a series of studies on the differences between the left and right hemispheres of the brain. For example, Li et al. proposed a bi-hemispheric discrepancy (BiHDM), which was used to learn the asymmetrical discrepancy between two hemispheres for emotion recognition by EEG [10].

Although deep learning has achieved good recognition accuracy for EEG-based emotion classification, there is still large room for improvement. This is because most studies use information from different frequency bands as input to extract frequency band information while ignoring the complementarity of information between different frequency bands, and thus cannot fully exploit the information in a single frequency band. To solve this problem, SFCSAN uses intra-frequency band self-attention to learn frequency information from each frequency band, uses a parallel convolutional neural network (CNN) to mine spatial information, and combines inter-band mapping to focus on the information of different frequency band subspaces to mine complementary information, and finally, the average accuracy of valence/arousal/dominance/like labels can reach 95.15%/95.76%/95.64%/95.86% on the DEAP dataset [11]. Inspired by this idea, we attempt to learn frequency-domain information from a single frequency band in parallel. Furthermore, to improve the deficiency of ignoring topology information when mining spatial information by CNN, we improve the extraction method of sub-band frequency-domain information and finally fuse the sub-band information according to the importance, allowing more spatial and frequency-domain information to be fully extracted. In addition, considering that not every electrode can play an important role in emotion classification and that there may be some redundancy between some electrodes, we further perform channel selection to reduce the time loss to ensure good model performance. Based on the above analysis, this study proposes an emotion classification method based on a dynamic simplifying graph convolutional (SGC) network and channel style recalibration module (SRM), termed SGC-SRM, with multi-band EEG data as input. The main contributions are as follows:

(1) A multilayer SGC network is built, which extracts sub-band features in parallel, updates the adjacency matrix through backpropagation, and realizes dynamic learning of EEG topology.
(2) A convolution layer based on SRM is introduced to recalibrate the channel features of the sub-band to improve emotion-related feature extraction.
(3) Features of four sub-bands are fused to achieve more accurate emotion classification, and 12 channels suitable for emotion classification are selected to reduce time consumption.

The remaining sections of this paper are arranged as follows: Section 2 gives an overview of SGC-SRM-related technologies. Section 3 describes the architecture and implementation of the proposed SGC-SRM model. In Section 4, experiments are conducted and the model performance and results are analyzed. Section 5 summarizes the main achievements of this study and highlights the future research direction.

## 2. Related Works

### 2.1. EEG Emotion Classification Based on Spatial-Domain

EEG-based emotion classification frequently uses CNNs to extract spatial information from EEG (e.g., EmotionNet [12]). Nevertheless, there is more redundant information between multi-channel signals, which not only increases time consumption but also reduces classification accuracy. To compensate for the shortcomings of CNNs, some researchers extract the relationship between different EEG signal channels through capsule networks. For example, Kumari et al. used the short-term Fourier transform algorithm to transform raw one-dimensional EEG signals into a two-dimensional spectrogram image and implemented a capsule network to process the spatio-temporal characteristics of EEG signals. The average accuracy of valence, arousal, and dominance on the DEAP dataset is 77.50%, 78.44%, and 79.38% respectively [13]. Deng et al. used a capsule network to extract the spatial features of EEG channels, combined with the attention mechanism to adaptively assign different weights to each EEG channel, and used LSTM to extract the temporal features of EEG sequences. The average accuracy of valence, arousal, and dominance on the DEAP dataset is 97.17%, 97.34%, and 96.50%, respectively [14]. However, the dynamic routing operation of capsule networks requires significant computational overhead; thus, it is necessary to find optimal solutions.

Graph neural networks (GNNs) were introduced in 2009 by Scarselli et al. to deal with graph data [15]. The improved graph CNN (GCNN) method combines CNNs with spectral theory and provides an effective method to describe the intrinsic relationships between different nodes of a graph [16]. Due to this, the spatial location connection between channels in EEG-based emotion classification does not represent the functional connection between them. Song et al. proposed a dynamic graph CNN (DGCNN), which used the Gaussian kernel function to initialize the adjacency matrix, and dynamically learned the internal relationship between different EEG channels represented by the adjacency matrix. On the SEED dataset, subject-independent accuracy reaches 79.95% [9]. Subsequently, a GNN has been extensively used for EEG-based emotion classification. For example, Song et al. proposed a graph-embedded CNN (GECNN) to extract distinctive local features, and global features were captured to identify EEG emotions [17]. Jin et al. proposed a graph convolutional network (GCN) with learnable electrode relations that learns the adjacency matrix automatically in a goal-driven manner using the two-dimensional distribution of electrodes as the initial adjacency matrix (0 indicates that two electrodes are not adjacent; 1 indicates that they are adjacent), and the subject-dependent recognition accuracy of DE features on SEED was 94.72% [18]. Li et al. proposed a self-organizing GNN (SOGNN) for cross-subject, where the graph structure was dynamically constructed using the self-organized module of each signal for EEG-based emotion classification [19]. Zhang et al. proposed a sparse DGCNN (SparseD) by imposing a sparseness constraint on the weighted graph to improve EEG-based emotion classification performance [20].

### 2.2. Channel Selection and Sub-Band Feature Extraction

EEG signals contain rich brain activity information and are distributed in different frequency bands [21]. Wang et al. found that emotional characteristics were mainly related to high-frequency bands, e.g., the alpha band is located in the right occipital lobe and parietal lobe, the beta band is located in the parietal and temporal lobes, and the gamma band is located in the left frontal lobe and right temporal lobe [22]. Therefore, the distribution of emotion-related information is not the same at different sub-bands. Zhu et al. proposed an EEG-based emotion classification network based on the attention fusion of multi-channel band features, which combined multiple frequency bands through feature addition, multiplication, and attention; the highest accuracy achieved on SEED was 96.45% [23]. Therefore, if the information of each sub-band is extracted in parallel and then the emotion-related information of each sub-band is fully mined by the importance fusion, classification performance will be improved.

During the collection of EEG signals, multiple electrodes are frequently used to cover the scalp surface to collect as many comprehensive EEG signals as possible. However, for emotion classification, not all electrodes can play an important role; the more electrodes, the larger the data, and the longer the emotion classification will take. Zheng et al. designed different electrode arrangements according to the features of high peaks in the weight distribution and asymmetric properties in emotion processing: 4 channels (“FT7”, “FT8”, “T7”, “T8”), 6 channels (“FT7”, “FT8”, “T7”, “T8”, “TP7”, “TP8”), 9 channels (“FP1”, “FPZ”, “FP2”, “FT7”, “FT8”, “T7”, “T8”, “TP7”, “TP8”), and 12 channels (“FT7”, “T7”, “TP7”, “P7”, “C5”, “CP5”, “FT8”, “T8”, “TP8”, “P8”, “C6”, “CP6”). Support vector machine classification produced good results on the 12 channels using DE characteristics, with average accuracy of 86.65% [7]. The time cost can be reduced without affecting accuracy as much as possible through channel selection.

### 2.3. SRM

SRM was proposed to adaptively recalibrate intermediate feature maps using the style information of the intermediate feature map [24]. Initially, an intermediate style representation T is extracted from each channel of the feature map X through style pooling, and then, the per-channel recalibration weight G is estimated through style integration independent of channels. Finally, the input features X and G are calculated to obtain the calibrated feature map X′.

**Figure 1:**
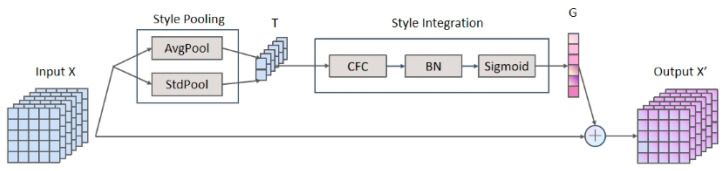
SRM structure diagram.

As depicted in Figure 1, SRM is mainly composed of style pool and integration: (1) the style pool is introduced to calculate the average and standard deviation of each feature map to extract style features T; (2) the style integration is composed of a channel-wise fully connected layer (FC), a batch normalization (BN) layer, and a sigmoid activation function.

Inspired by image style migration, SRM was originally used to extract image style and incorporate relatively important style features into feature maps. Zhang et al. designed a style discriminator with an SRM to capture seasonal style features on remote sensing images [25]. Lu et al. performed target detection on video-induced EEG signals, extracted EEG spatio-temporal features with graph convolution, and improved the SRM to select features with larger contributions [26]. Bao et al. added an SRM to a CNN to extract deep features and select features with high correlation with emotion and obtained improved results in a subject-depended experiment on the SEED dataset (95.08%) [27]. Therefore, the introduction of the SRM to adaptively recalibrate the intermediate features learned from sub-bands can incorporate them into the feature maps, thereby minimizing the loss of information and improving the feature extraction ability of the network [27].

## 3. Methodology

To effectively utilize topology information of EEG signals in both the frequency and spatial domains, the SGC-SRM model is proposed in this study. First, the DE feature extracted from each sub-band of an EEG signal is used as input. Second, considering that different emotional states show different degrees of activation in different frequency bands [6], we extract the features of each sub-band respectively, and then fuse them according to the importance of the frequency bands, which can better mine the information in the frequency-domain. Given that EEG signals contain topological information, we improve the dynamic SGC to learn the relationship between channels. When extracting sub-band features, we added an SRM-based convolution layer to adaptively learn the intermediate feature map and recalibrate the channel features to emphasize the information related to emotions and ignore other information. Finally, we use the full connection layer and softmax for the triple classification (positive, neutral, negative).

**Figure 2:**
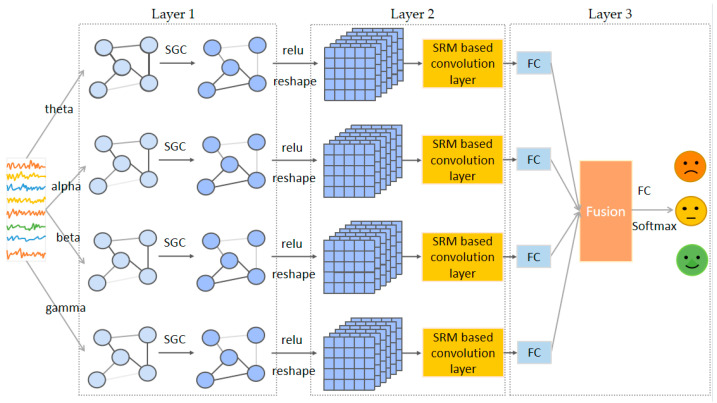
Framework diagram of SGC-SRM.

The framework of the proposed SGC-SRM model is depicted in Figure 2, which consists of three layers: (1) Layer 1: Dynamic SGC layer; (2) Layer 2: SRM convolution layer; (3) Layer 3: Fusion and classification layer.

### 3.1. Layer 1: Dynamic SGC Layer

#### 3.1.1. Construction of Adjacency Matrix

The definition of EEG in the graph structure is represented by G = (V, E, A); among them, V denotes the set of nodes of the graph, |V| = C; E represents the set of edge connections between different nodes; A represents a symmetric adjacency matrix with A∈R^N×N^, A_ii_ = 1; and the elements a_ij_ of A represent the edge weights between nodes vi and vj, which are used to represent the relationship between EEG channels. Salvador et al. found that local brain correlations typically decay as the Euclidean distance between the centroids of regions increases, and this nonlinear relationship can be approximately described by an inverse square law [28]. We refer to the adjacency matrix definition of a regularized GNN (RGNN) [29]:

(1) $$A_{ij}=\min\left(1,\frac{\delta }{d_{ij}^{2}}\right)$$

where d_ij_ denotes the physical distance calculated from the 3D coordinates of channels i and j on the device that collects EEG signals, and δ = 5, ensuring that approximately 20% of the relationship between channels is not ignored [29].

To take advantage of DASM information, we introduce several global connections: (FP1, FP2), (AF3, AF4), (F5, F6), (FC5, FC6), (C5, C6), (CP5, CP6), (P5, P6), (PO5, PO6), (O1, O2). By changing the initialization of the adjacency matrix elements corresponding to these connections [29], we also testify that global connections play a certain role in the model through the ablation experiments in the subsequent chapters.

#### 3.1.2. SGC Network

SGC (simplifying GCN) is a variant of GCNs. GCNs were first proposed by Kipf and other authors [30]. Like CNNs and multilayer perceptrons, the eigenvectors of each node through multilayer networks are first learned by GCNs, and then these eigenvectors are used as input to linear classifiers. The difference between GCNs and multilayer perceptrons is that the hidden representation of each node is the average of its neighbors at the beginning of each layer. A graph convolutional layer contains three strategies for updating node representations: feature propagation, linear transformation, and a nonlinear activation layer. The transmission mode between GCNs layers is represented as follows:

(2) $$H^{\left(K+1\right)}=\sigma \left({\mathrm{SH}}^{\left(K\right)}\Theta^{\left(K\right)}\right)$$

(3) $$S={\tilde{D}}^{-\frac{1}{2}}\tilde{A}{\tilde{D}}^{-\frac{1}{2}}$$

where S represents the normalization of the adjacency matrix A; $\tilde{A}=A+I$, I represents the identity matrix; $\tilde{D}\;$represents the degree of matrix $\tilde{A}$; H represents the feature of each layer; and σ represents the nonlinear activation function, which is H_0_ for the input layer; K is an integer, representing the number of layers. To reduce the excessive complexity of GCNs, Wu et al. proposed SGC, which iteratively removes nonlinearities between GCN layers and collapses the generated function into a single linear transformation. Experiments show that SGC is more computationally efficient than GCNs while being able to show comparable or even better performance [31]. The propagation mode between SGCs layers can be expressed as follows:

(4) $$H^{\left(K+1\right)}=S^{K}X\Theta$$

where X represents the input; S^K^ = SS … S represents the repeated multiplication of the normalized adjacency matrix S into a single matrix; Θ = Θ^(1)^Θ^(2)^…Θ ^(K)^ means that the weights are reparameterized into a single matrix.

#### 3.1.3. Improved Dynamic SGC

Studies have shown that DE features have stronger discriminative power in emotion recognition than other features [7,9,19]. Therefore, the DE features of EEG signals are used as the input of the model in this study, For X~N(μ, σ^2^), DE features are calculated as follows:

(5) $$H\left(x\right)=-\int_{-\infty }^{\infty }\frac{1}{\sqrt{2{\pi \sigma }^{2}}}\exp\frac{{\left(x-\mu \right)}^{2}}{2\sigma^{2}}\log\frac{1}{\sqrt{2{\pi \sigma }^{2}}}\exp\frac{{\left(x-\mu \right)}^{2}}{2\sigma^{2}}\mathrm{dx}=\frac{1}{2}\log2{\pi e\sigma }^{2}$$

Then, the input of the model is denoted as X∈RN × C × B × D; labels Y∈ZN; N represents the number of samples; C denotes the number of channels; B denotes the number of frequency bands; D denotes the feature dimension. The proposed model first initializes an adjacency matrix Ab to be the DE features Xb of each sub-band (see the descriptions in Section 3.1.1), where Xb∈RN × C × b × D, b∈{δ, θ, α, β, γ} or b∈{θ, α, β, γ}. Subsequently, the adjacency matrix Ab is taken as the input of the dynamic SGC layer. We note that in the dynamic SGC layer, the SGC (see Section 3.1.2) is performed twice. During each time, K is set to be one (i.e., the feature representation of a node is derived by information aggregation of its neighbor nodes), the size of the features is changed by each convolution operation, and an activation function is added to the last layer.

The model constructs a graph structure for each sub-band, and then extracts the sub-band features separately (in parallel) and performs fusion, that is, an adjacency matrix is constructed for each frequency band; finally, the cross entropy loss (function) of the RGNN [29] is improved. To be specific, if the total number of frequency bands is B, then the number of adjacency matrixes constructed is B and the improved loss function is as follows:

(6) $$\Phi =\mathrm{CrossEntropy}+a\times \sum_{i}^{B}∥A_{b}{∥}_{1}\;$$

where B represents the total number of frequency bands; A_b_ represents the adjacency matrix constructed from the data of band b; a represents the L1 regularization strength of the adjacency matrix, and the value of a is 0.01 (see Section 4.3.5 for the value analysis of a). CrossEntropy represents the crossentropy loss function, and its formula is as follows:

(7) $$H\left(p,q\right)=-\frac{1}{N}\sum_{i=1}^{N}p\left(x_{i}\right)\log\left(q\left(x_{i}\right)\right)$$

where $p\left(x_{i}\right)\;$represents the true one-hot encoding vector and $q\left(x_{i}\right)\;$represents the predicted encoding vector.

Due to the fact that the graphic structure is fixed, it cannot simulate the states of different subjects under different emotions. Therefore, by calculating the gradient of the loss function with respect to A, the adjacency matrix A is dynamically updated using the backpropagation algorithm to dynamically learn the relationship between channels, as shown in the formula:

(8) $$A_{b}\leftarrow A_{b}-\mathrm{lr}\left(\frac{\partial \Phi }{\partial A_{b}}\right)$$

where lr represents the learning rate.

### 3.2. Layer 2: SRM Convolution Layer

**Figure 3:**
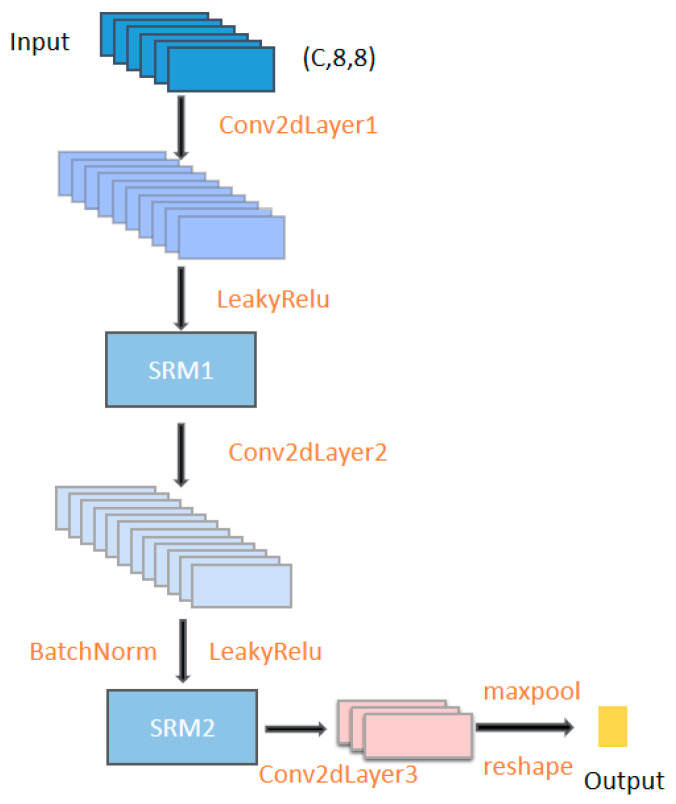
Framework diagram of convolutional layers based on SRM.

The input of the SRM convolutional layer [24] is a deformation of the dynamic SGC output of Section 3.1.3, which is encoded into the feature space using three convolutional layers and two SRM layers. The specific process is depicted in Figure 3 (see Table 1 for the parameters), which can assign a large weight to important features in the sub-band and a small weight to features weakly correlated with emotion.

### 3.3. Layer 3: Fusion and Classification Layer

During feature fusion, the model adaptively learns the weights of feature maps extracted from different sub-bands, thereby improving the classification ability of the model. Assume that there are parameters of B frequency bands that can be learned. Wi is defined to represent their weights, and $\overset{4}{\underset{i=1}{\sum }}W_{i}$ = 1. The information of four sub-bands is fused to obtain the following:

Band_f_ = W_1_ × Band_θ_ + W_2_ × Band_α_ + W_3_ × Band_β_ + W_4_ × Band_γ_ (9)

where Band_f_ represents the fused feature; Band_θ_, Band_α_, Band_β_, and Band_r_ denote the DE features on the bands of θ, α, β, and r, respectively. Finally, a fully connected layer and softmax are used for emotion classification.

## 4. Experiments

### 4.1. Dataset

The experiment was conducted on the public dataset SEED, which is an EEG emotion dataset released by Shanghai Jiaotong University. Fifteen subjects participated in the experiment, with each subject participating in three sessions of experiments, and each group watched 15 movie clips, with a total of 675 samples [7,32]. In this study, DE features from the dataset are used as input to the model. The dataset provider uses a non-overlapping Hamming window with a window length of 1 s and short-time Fourier transform to extract five frequency bands of EEG signals (delta: 1–3 Hz; theta: 4–7 Hz; alpha: 8–13 Hz; beta: 14–30 Hz; gamma: 31–50 Hz); then, DE features are calculated. To normalize the processing period, we refer to the self-organized GNN (SOGNN) model and fill the SEED data window to 265 with zero if the window is less than 265 [19].

### 4.2. Experimental Setup

Generally, the verification strategy based on EEG-based emotion classification has two forms: subject-dependent and subject-independent. On the public benchmark dataset SEED, this study uses a leave-one-subject-out (LOSO) cross-validation method to evaluate the performance of the model and a subject-independent strategy to evaluate the ability of the model to recognize “strange” subjects’ emotions. Specifically, the DE features of 14 subjects are used as the training set, whereas the data of the remaining subject are used as the test set. Fifteen-fold experiments are conducted. As the training set, each fold contains data from different subjects. After the model converges, the average value of the last 10 epochs is taken as the experimental result of each fold [33]. The final evaluation result of the model is the average accuracy (ACC) and standard deviation (STD) of the results obtained for each fold. Before training, we normalize the data of each subject, that is, subtract the average value from the characteristics of each subject and then divide it by its STD [19].

The model is trained on NVIDIA GeForce GTX 1080 Ti, the initial value of the learning rate is 0.001, and the learning rate is dynamically adjusted with exponential decay. The epoch of this experiment is 50, the optimization function is set to Adam optimization, and the batch size is 64.

### 4.3. Scheme Validation

According to the structure of the SGC-SRM model, the experiment needs to verify the effectiveness of the following scheme:

(1) Whether the performance of multi-bands is better than that of a single band.
(2) Whether the features of the fused sub-bands are better than those of all bands directly extracted or not.
(3) Whether channel selection can decrease time consumption.
(4) Whether the DE feature is the most effective.
(5) Whether the selection of parameter a (L1 sparse regularization strength) is effective or not.

#### 4.3.1. Band Performance

(1) Performance of single band

When the fusion layer between sub-bands of the SGC-SRM model is eliminated, the full connection layer and softmax activation function are directly used for classification (named Band-SGC-SRM) to verify the impact of a single band on emotion. As presented in Table 2, when taking the DE feature of one of the {δ, θ, α, β, γ} bands as input, the final results (ACC ± STD) are 89.45% ± 6.15%, 92.93% ± 4.09%, 87.90% ± 7.29%, 90.87% ± 7.03%, and 91.54% ± 6.18%, respectively.

(2) Performance of multi-bands

Because four of the five frequency bands of EEG signals are closely related to human emotions [34,35], considering that many studies use the five frequency bands commonly used, we develop two data input methods: Four bands = {θ, α, β, γ}, indicating that the DE feature data extracted from these four bands θ, α, β, γ will be used as input; all bands = {δ, θ, α, β, γ}, indicating that the DE feature data extracted from the five bands δ, θ, α, β, γ will be used as input.

As shown in Table 2, when the data of four bands or all bands are used as the input of the model Band-SGC-SRM, the classification result (ACC ± STD) is 94.07% ± 4.11% or 93.78% ± 4.23%, respectively, which outperforms single band performance in (1).

(3) Performance of sub-band feature fusion

The data of four bands or all bands are used as the input of the SGC-SRM model, an adjacency matrix is constructed for each sub-band, and the features of each sub-band are extracted in parallel. Subsequently, fusion classification is further performed using LOSO cross-validation strategy, and its results are shown in the last two rows of Table 2. For clarification, in the case when using four bands as input, called Fusion (θ, α, β, γ), the resulting average accuracy is 94.77% and STD is 4.48%; while in the case when using all bands as input, called Fusion (All bands), the resulting average accuracy is 94.90% and STD is 3.94%. This indicates that the sub-band feature fusion strategy proposed in this study has better performance than the results of not fusing sub-bands in (2).

#### 4.3.2. Channel Selecting Performance

**Figure 4:**
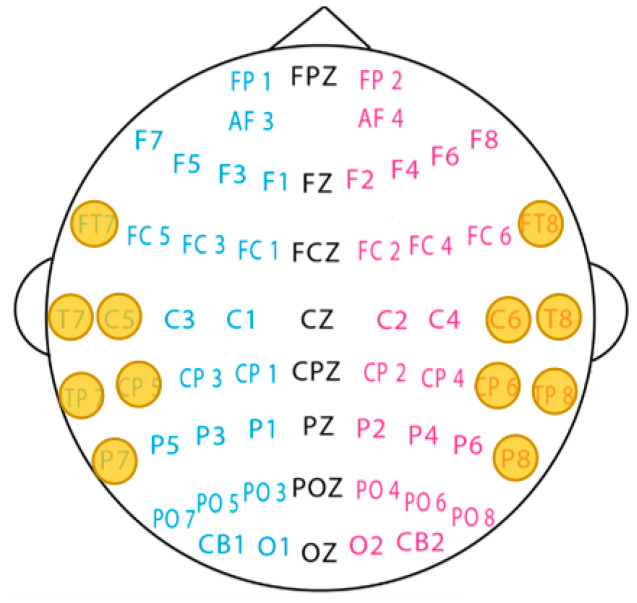
Channel selection (the proposed 12 channels are marked with yellow circles).

As shown in Figure 4, 12 channels of “FT7”, “T7”, “TP7”, “P7”, “C5”, “CP5”, “FT8”, “T8”, “TP8”, “P8”, “C6”, and “CP6” are selected in this study [7]. Using the same settings as in Section 4.3.1, we conduct experiments of sub-band fusion and direct extraction of features from all frequency bands based on four and five frequency bands, respectively. As presented in Table 2, we can conclude the following: (1) the results of 12 channels are consistent with the results of 62 channels; (2) the performance of applying four- and five-band features is comparable; (3) fusing the features of sub-bands is better than directly extracting the features of all bands.

**Figure 5:**
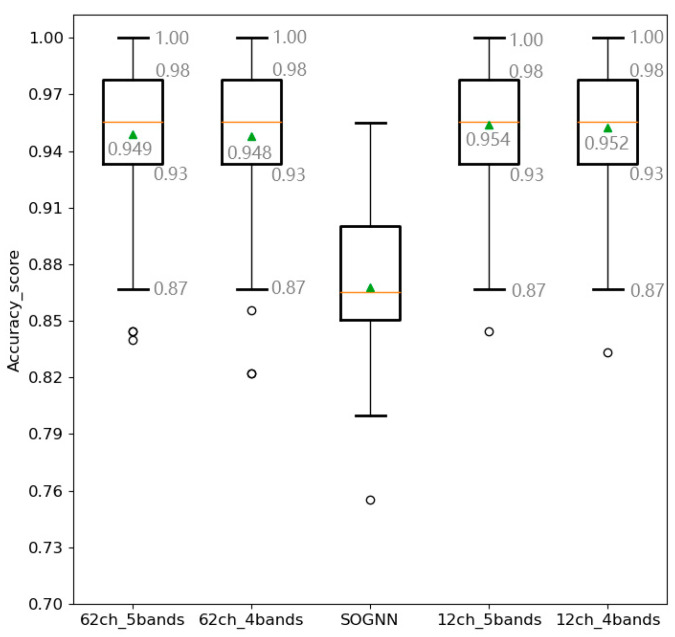
Box plot of multi-band feature fusion of different channels.

To further evaluate the classification performance of different multi-band and multi-channel methods, we conduct experiments and display the box diagram in Figure 5. 

In the middle of Figure 5, SOGNN [19] is introduced as a baseline using the DE features of five frequency bands in the SEED dataset and the box plot obtained by the LOSO cross-validation. From this figure, we can see that the maximum, median (orange horizontal line in the figure), average (green triangle), minimum, upper quartile (upper rectangular line, indicating that 25% of the number is greater than the value), and lower quartile (lower rectangular line, indicating that 25% of the number is less than the value) of all fold results of the SGC-SRM model are better than those of SOGNN. From the comparison between using four and five sub-bands feature fusion in Figure 5, we can see that (i) The maximum, minimum, upper quartile, and lower quartile are the same in the cases of using 62 and 12 channels; (ii) The average of using 12 channels is slightly better than that of using 62 channels.

#### 4.3.3. Time Consumption

**Figure 6:**
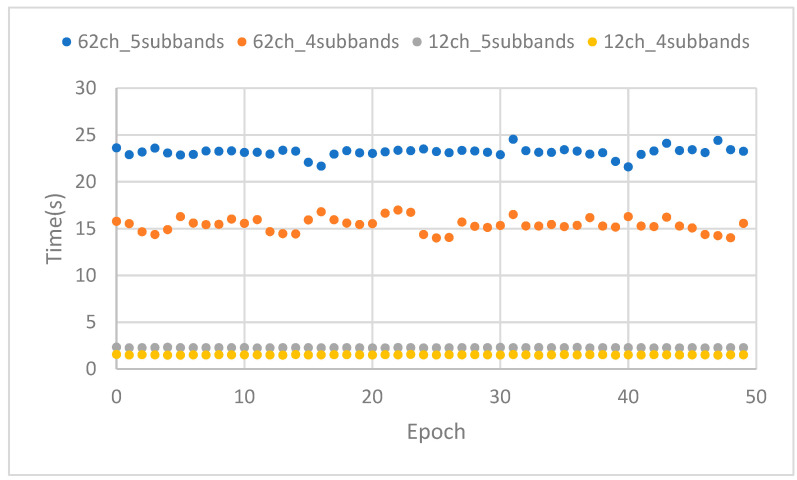
Time overhead comparison graph.

To more intuitively observe the impact of the number of channels and frequency bands on the calculation time, a fold experiment with a cross-validation experiment is randomly selected, and the times required for different channel numbers in four and five sub-bands are compared, as shown in Figure 6. We note that, for the 62 channels, the total time required to apply five sub-bands is 17,394.64 s; in contrast, the total time required for four sub-bands is 11,525.26 s, which is 33.74% lower than the former. For the 12 channels, the total time required to apply five and four sub-bands are 1494.39 and 1146.19 s, respectively. The latter reduces the operation time by 23.30%. Moreover, compared with using 62 channels, applying five sub-bands and 12 channels can save 91.41% of the total time; applying four sub-bands with 12 channels can save 90.5% of the total time.

Combining Section 4.3.1, Section 4.3.2 and Section 4.3.3, the following conclusions are obtained:

(1) The time for feature fusion when using five sub-bands (the average of three groups of experiments) is significantly higher than that when using four sub-bands, but with equivalent performance. Specifically, using five bands is slightly better than four bands, with an accuracy difference of 0.13% for 62 channels and 0.16% for 12 channels, see Table 2.
(2) The accuracy of using 12 channels is slightly better than that of using 62 channels. From the box plot (the green triangle symbol is the average value) in Figure 5, the average accuracy of using 12 channels is slightly higher than that of using 62 channels. Compared with the box plot of SOGNN, the SGC-SRM model is better than SOGNN in terms of the maximum value, minimum value, average value, and median.
(3) In terms of time consumption, using 12 channels is significantly better than 62 channels, see Figure 6.

Therefore, selecting four sub-bands DE features of 12 channels is suitable for emotion classification because of better performance and lower time consumption.

#### 4.3.4. Effectiveness of DE Characteristics

**Figure 7:**
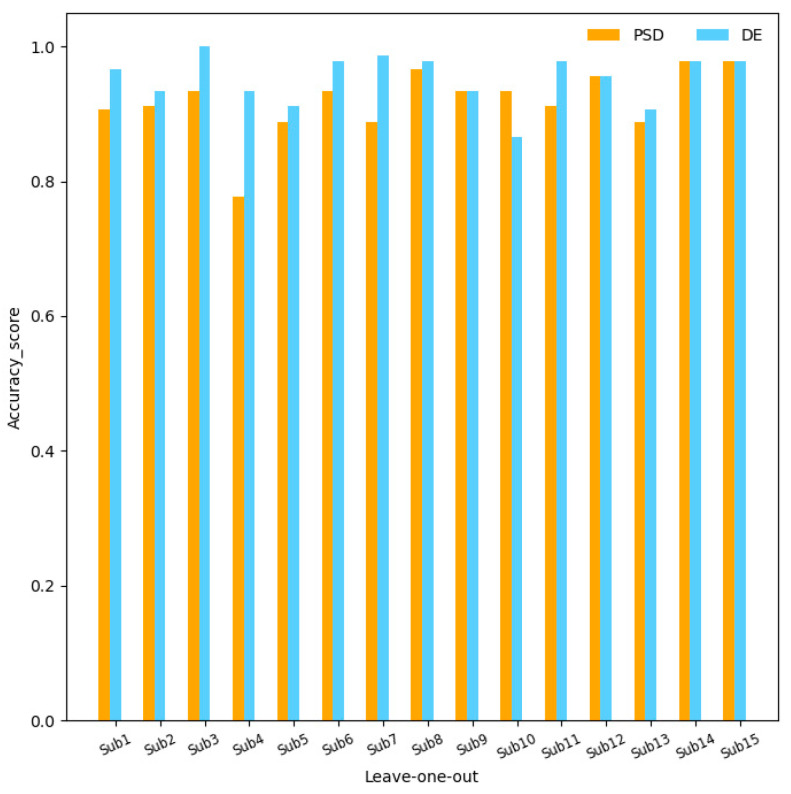
Comparison of PSD and DE characteristics.

To verify the effectiveness of the DE features, we use the 12 channels and four sub-bands optimized as the input of the SGC-SRM model and obtain 15 rounds of cross-validation results using DE and PSD features, as depicted in Figure 7. The average accuracy and standard deviation obtained by applying PSD features are 91.90% ± 4.78%; the average accuracy and standard deviation of DE features are 95.22% ± 3.61%. The results show that the application of DE features has higher accuracy and lower standard deviation than traditional PSD features, which is consistent with the research conclusion of [8].

#### 4.3.5. Effectiveness of L1 Sparse Regularization Strength Parameter (a)

**Figure 8:**
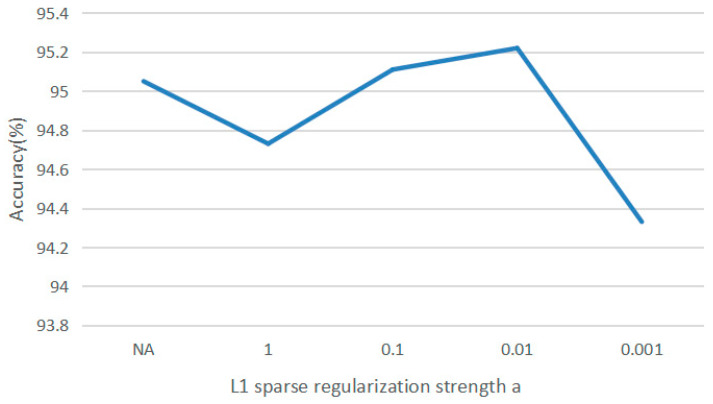
Comparison of different parameters of a.

For the choice of parameter a, we choose a = {1, 0.1, 0.01, 0.001} for the experiments, and the obtained results are shown in Figure 8, where a = 0.01 exhibits the best performance. To further verify the effectiveness of the improved loss function, a × $\overset{m}{\underset{i}{\sum }}∥A_{b}{∥}_{1}$ is removed from the loss function, the adjacency matrix is a fixed matrix and will not change dynamically, and “NA” in the figure means that a × $\overset{m}{\underset{i}{\sum }}∥A_{b}{∥}_{1}$ is removed from the loss function; namely, the adjacency matrix is a fixed matrix. The results show that backpropagation dynamically changes the adjacency matrix, which can sometimes better extract the spatial relationship of EEG signals.

### 4.4. Model Overall Performance and Result Analysis

#### 4.4.1. Comparative Experiment

To further evaluate the overall performance of the SGC-SRM model, we conduct a series of experiments on the public dataset SEED. The relevant baseline methods, which focus on the influence of sub-bands on emotion classification using DE features and the LOSO cross-validation strategy, are listed as follows. The results are shown in Table 3.

DGCNN [9]: Multi-channel EEG-based emotion classification method based on DGCNNs that initializes the adjacency matrix and trains the adjacency matrix dynamically through backpropagation.

GECNN [17]: A deep learning method used for EEG emotion recognition, where the CNN is used to extract different depth local features, and then dynamic graph filtering is used to explore the internal relationship between different EEG regions.

BiDANN-S [36]: A deep learning method used for EEG-based emotion classification, where the original EEG features extracted from each cerebral hemisphere are used to extract differentiated depth features, and domain discriminators are used to alleviate domain differences between the source and target domains.

BiHDM [10]: A bi-hemispheric discrepancy model learns asymmetrical differences between two hemispheres, using four recurrent neural networks to capture information from EEG electrodes in each hemisphere from horizontal and vertical streams.

RGNN [29]: A regularized GNN for EEG-based emotion classification, extended SGC, which uses the adjacency matrix to model channel relationships in EEG signals. To effectively deal with cross-subject EEG variations and noisy labels, node-wise domain adversarial training and emotion-aware distribution learning are proposed.

SOGNN [19]: A self-organizing GNN for cross-subject emotion classification of EEG, which dynamically constructs the graph structure according to the corresponding EEG features of the input and processes the graph structure by three graph convolution layers to extract the local and global connection features for emotion recognition.

SparseD [20]: A sparse DGCNN model, which introduces sparse constraints into the graph representation to improve the DGCNN.

As shown in Table 3, adopting the LOSO cross-validation strategy, with the DE features of four frequency bands as input, the average accuracy of the SparseD model is 89.71% (62 channels were applied), and that of SGC-SRM is 95.22% (12 channels are applied). When DE features of five frequency bands are used as input, the average accuracy of the DGCNN, GECNN, BiDANN-S, BiHDM, RGNN, SOGNN, and SparseD models using 62 channels are 79.95%, 82.46%, 84.14%, 85.40%, 85.30%, and 86.81%, respectively. In contrast, the average accuracy of the SGC-SRM model using 12 channels is 95.38%, which indicates that the proposed model can obtain better classification accuracy by using fewer channels (i.e., a small amount of data).

#### 4.4.2. Ablation Experiments

To verify the effectiveness of important modules in the SRM-SGC model, we conducted a series of ablation experiments on the SEED dataset, including the following:

(1) Verify the effectiveness of global connection on 62 channels or 12 channels.
(2) Verify the effectiveness of the convolution layer without SRM on 62 channels or 12 channels.
(3) Verify the effectiveness when removing global connections and SRM-based convolutional layers simultaneously.

The results are shown in Table 4. If the global connection is removed, the average accuracy of the model decreases by 0.99% on 62 channels and 0.43% on 12 channels; please see the comparison between the first two rows of Table 4. After removing the SRM-based convolutional layer, the average accuracy decreases by 4.24% on 62 channels and 1.77% on 12 channels; see the first and third rows of Table 4. When both global connections and SRM-based convolutional layers are removed, the average accuracy drops by 4.73% on 62 channels and 1.74% on 12 channels; see the first and fourth rows of Table 4. The results show that global connection can enhance the information of learning asymmetric channels and improve the performance of the model. Introducing a convolutional layer based on SRM and recalibrating the channel features of sub-bands can effectively improve the ability of emotion-related feature extraction, thereby improving the model’s classification accuracy.

#### 4.4.3. Confusion Graph of SGC-SRM

**Figure 9:**
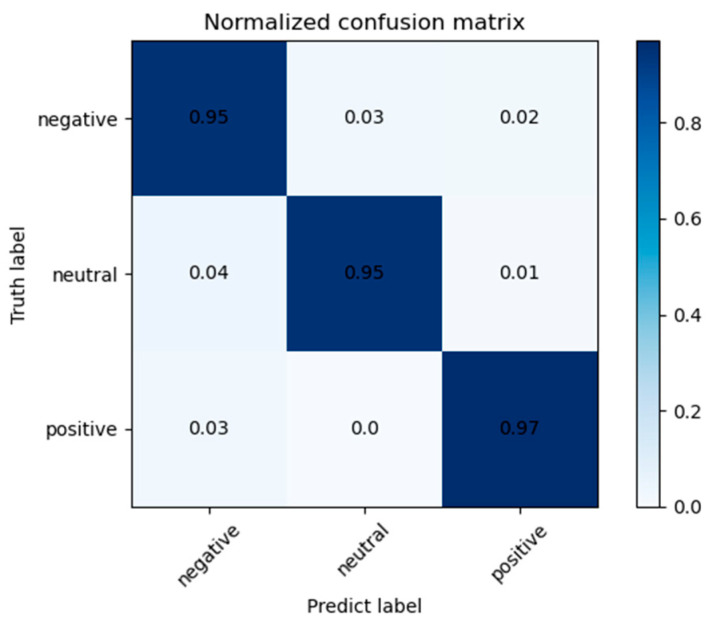
Confusion matrix for LOSO cross-validation.

To further verify the performance of the SGC-SRM model, the confusion matrixes of LOSO are shown in Figure 9. The horizontal axis represents the prediction label of the model, and the vertical axis represents the actual label of the model. The three category labels are negative, neutral, and positive from left to right and from top to bottom. The SGC-SRM model exhibited good results in identifying negative, neutral, and positive emotions in the SEED dataset. The results obtained by LOSO cross-validation were 95%, 95%, and 97%, respectively. Among the emotions identified as wrong, those labeled neutral are easier to be identified as negative emotions. The probability of such errors is 4% for LOSO cross-validation. Happy emotions are more easily identified than neutral and negative emotions.

## 5. Conclusions

In this study, we propose an EEG-based emotion classification method based on multi-band dynamic SGC and channel feature recalibration. A multilayer SGC is constructed to learn sub-band features in parallel, and a convolution layer based on SRM is introduced to recalibrate channel features. In addition, 12 channels suitable for emotion classification are selected to save time. Furthermore, the performance of single band, multi-band direct input, and sub-band feature fusion is compared, and the results show that the proposed sub-band feature fusion can achieve high-accuracy emotion classification. In addition, ablation experiments verify the effectiveness of important layers in our model. In the future, the SGC-SRM model will be applied to other physiological signals or fused with various non-physiological signals to improve the accuracy of emotion classification.

## Figures and Tables

**Table 1:** Parameters of the convolutional layer based on SRM.

Stage	Kernel	Stride	Input	Output
Conv2dLayer1	2	1	(N,C,8,8)	(N,out1,7,7)
SRM1	-	-	(N,out1,7,7)	(N,out1,7,7)
Conv2dLayer2	2	1	(N,out1,7,7)	(N,out2,6,6)
SRM2	-	-	(N,out2,6,6)	(N,out2,6,6)
Conv2dLayer3	2	1	(N,out2,6,6)	(N,out3,6,6)
Maxpool2d	2	1	(N,out3,6,6)	(N,out3,3,3)

Note: when there are 62 channels: out1 = 128; out2 = 256; out3 = 8. 12 channels: out1 = 32; out2 = 64; out3 = 8. N represents mini-batch; C represents the number of channels. “-” indicates that the parameter is not provided.

**Table 2:** Channel selection and band performance (ACC ± STD).

Frequency Band	SEED	
	62ch	12ch
δ	89.45 ± 6.15	91.35 ± 5.12
θ	92.93 ± 4.09	92.47 ± 4.28
α	87.90 ± 7.29	83.48 ± 8.10
β	90.87 ± 7.03	90.96 ± 5.10
γ	91.54 ± 6.18	92.03 ± 4.54
θ, α, β, γ	94.07 ± 4.11	94.25 ± 4.74
All bands	93.78 ± 4.23	94.34 ± 3.77
Fusion (θ, α, β, γ)	94.77 ± 4.48	95.22 ± 3.61
Fusion (All bands)	**94.90 ± 3.94**	**95.38 ± 3.51**

Note: 62ch in the table represents 62 channels when the SEED dataset is collected. Figure 4 shows the detailed locations and names of 62 channels; 12ch means 12 channels: “FT7”, “T7”, “TP7”, “P7”, “C5”, “CP5”, “FT8”, “T8”, “TP8”, “P8”, “C6,” and “CP6.”.

**Table 3:** Leave-one-subject-out emotion recognition accuracy (mean ± standard deviation) on SEED.

	Band	SEED						
Method		δ	θ	α	β	γ	θ, α, β, γ	All Bands
DGCNN		49.79 ± 10.94	48.29 ± 12.28	48.29 ± 12.28	56.15 ± 14.01	54.87 ± 17.53	/	79.95 ± 9.02
GECNN		62.11 ± /	63.60 ± /	61.79 ± /	75.28 ± /	75.41 ± /	/	82.46 ± /
BiDANN-S		63.01 ± 07.49	63.22 ± 07.52	63.50 ± 09.50	73.59 ± 09.12	73.72 ± 08.67	/	84.14 ± 6.87
BiHDM		/	/	/	/	/	/	85.40 ± 7.53
RGNN		64.88 ± 06.87	60.69 ± 05.79	60.84 ± 07.57	74.96 ± 08.94	77.50 ± 08.10	/	85.30 ± 6.72
SOGNN		70.37 ± 7.68	76.00 ± 6.92	66.22 ± 11.52	72.54 ± 8.97	71.70 ± 8.03	/	86.81 ± 5.79
SparseD		/	77.30 ± 10.08	79.29 ± 12.95	90.62 ± 12.08	90.65 ± 10.93	89.71 ± 11.92	/
SGC-SRM		**91.35 ± 5.12**	**92.47 ± 4.28**	**83.48 ± 8.10**	**90.96 ± 5.10**	**92.03 ± 4.54**	**95.22 ± 3.61**	**95.38 ± 3.51**

Note: “/” indicates that the literature is not provided.

**Table 4:** Ablation experiments.

	Channel	SEED	
Model		62ch	12ch
SGC-SRM		**94.77 ± 4.48**	**95.22 ± 3.61**
-global		93.78 ± 4.75	94.79 ± 3.08
-SRM		90.53 ± 5.06	93.45 ± 4.89
-global-SRM		90.04 ± 7.63	93.48 ± 4.90

Note: -global: Represents the removal of global connections. -SRM: Represents the removal of the SRM-based convolution layer. -global-SRM: Represents the removal of global connectivity and SRM-based convolution layers.

## Data Availability

The open access dataset SEED is used in our study. Its links is as follows, https://bcmi.sjtu.edu.cn/~seed/seed.html (granted on 7 May 2020; accessed on 25 April 2022).
